# Assessing the reliability and validity for metabolic syndrome diagnosis using point-of-care analyzers in a mobile setup - a pilot study

**DOI:** 10.1186/s12875-026-03502-3

**Published:** 2026-08-19

**Authors:** Sanne Houtenbos, Anne Schraplau, Pia-Maria Wippert, Michael Rapp, Heinz Völler, Klaus Bonaventura, Frank Mayer

**Affiliations:** 1 Medical Sociology and Psychobiology, Faculty of Human Sciences, University of Potsdam, Am Neuen Palais 10, Potsdam, 14469 Germany; 2 Sports Medicine and Sports Orthopedics, University Outpatient Clinic, University of Potsdam, Potsdam, Germany; 3 Social and Preventive Medicine, Faculty of Human Sciences, University of Potsdam, Potsdam, Germany; 4 Rehabilitation Medicine, University of Potsdam, Potsdam, Germany; 5 Klinik am See, Rehabilitation Center for Internal Medicine, Rüdersdorf, Germany; 6 Department of Cardiology, Ernst von Bergmann Hospital, Potsdam, Germany; 7 Faculty of Health Sciences Brandenburg, Joint faculty of the University of Potsdam, the Brandenburg Medical School Theodor Fontane and the Brandenburg University of Technology Cottbus-Senftenberg, Potsdam, Germany

**Keywords:** HbA1c, Mobile examination, Metabolic risk factors

## Abstract

**Background:**

The metabolic syndrome (MetS) is characterized by having three or more of these risk factors: increased blood glucose (Glc), serum triglycerides (TG), blood pressure (BP), waist circumference (WC), and reduced high-density lipoprotein (HDL). Mobile diagnostics using point-of-care (POC) blood analyzers could help identify individuals at risk. This pilot study assessed reliability and validity of MetS risk factor analysis using POC analyzers in a mobile examination unit.

**Methods:**

Fifty participants (52 ± 7y; 170 ± 10 cm; 80 ± 19 kg, m/f: 21/29) were included for analysis. The mobile examination unit was equipped with different POC analyzers (Alere, LABGEO, CardioChek for Glc, HDL, TG; Quo-Test-A1C Testkit for HbA1c‘). Agreement compared to a reference laboratory (RefLab) was analyzed by Bland-Altman (bias, Limits of Agreement (LoA)), ICC, SEM and McNemar’s test. Test-retest analysis was assessed on two different days (max. two weeks apart) using Spearman’s rho, ICC, test-retest variability (TRV%) and McNemar’s test.

**Results:**

Based on the smallest bias and low number of missing values (Glc: -5 [LoA − 25 to 6] mg/dl; TG: 8 [LoA − 58 to 30] mg/dl; HDL: -2 [LoA − 14 to 9] mg/dl), and the highest agreement in MetS diagnosis compared to the results of RefLab, the Alere POC device was chosen. Test-retest reliability (*n* = 16) for MetS risk factors and HbA1c measured in the mobile setup ranged from poor to excellent with low TRV (0.9–5.8%). Risk factor classification and MetS diagnosis did not differ between measurement days.

**Conclusions:**

A mobile setup using the Alere POC analyzer could be used as a valid mobile screening method for MetS to ensure early detection of risk factors.

## Background

The metabolic syndrome (MetS) is a worldwide public health problem [1]. The main concerns associated with MetS are a higher risk of type 2 diabetes mellitus, cardiovascular diseases and, possibly, mortality [1–8]. The MetS prevalence is approximately 19.8% among the German population [9, 10]. According to a review and modelling analysis by Noubiap et al. (2025) the prevalence of MetS increased globally from 2000 to 2023 from 14.7% to 31.0% among women and from 9.0% to 25.7% among men [11]. MetS is characterized by hyperglycemia, elevated serum triglycerides, decreased serum high-density-lipoprotein cholesterol (HDL), arterial hypertension, and increased waist circumference [1]. The economic burden due to complications related to MetS, such as cardiovascular diseases and obesity-related complications, is substantial [12]. The incidence and prevalence of MetS and its risk factors can be influenced by changing lifestyle factors, especially by increasing physical activity and improving diet [1, 6, 13]. To prevent the development of secondary diseases and reduce the disease and economic burden, early detection of MetS is necessary. With early detection, measures can be taken prohibiting MetS from progressing to further cardiovascular diseases.

Rural areas commonly have a higher burden of disease than urban regions. For example, the federal state of Brandenburg (Germany), which is a rural area with limited infrastructure, has one of the highest prevalences of MetS (27.5%) [10] and also of type 2 diabetes mellitus [14, 15] nationwide. As rural areas are often characterized by limited infrastructure and therefore lower access to health care providers as well as an acute shortage of physicians, different strategies and concepts to improve rural healthcare, such as telemedicine approaches and home visits by medical assistants, are currently subject of discussion and evaluation [16–18].

A fast, patient-centered, and decentralized approach is needed to reach people outside of central medical facilities and to identify risk profiles for diseases, such as MetS, even before the onset of symptoms and disease manifestation in order to enable early primary and secondary prevention. Such a concept could be realized through the use of mobile examination units for risk factor screening. Similar setups are already known for detecting other diseases, e.g. for diabetes diagnosis and for breast cancer screening with mammography [19–21]. For MetS screening, vehicles (vans) could be equipped, corresponding to medical examination rooms, that could be easily placed in rural areas to allow healthcare professionals to perform a brief on-site examination for the presence of a risk profile. Point-of-care (POC) analyzers must be part of such mobile units for the diagnosis of MetS, to provide fast, valid and reliable measurement results. POC devices help to reduce the time for analysis from hours or days (as in central laboratories) to minutes [22]. The use of POCs in a time- and cost-efficient approach can enable faster diagnosis, which can in turn contribute to early treatment or rapid implementation of prevention strategies. In recent years, a variety of devices for the analysis of blood parameters outside central medical facilities have been developed and validated. With the purpose of testing the concept of mobile diagnostics for early detection of MetS, it is first necessary to develop a mobile examination unit including suitable POC devices in combination with anthropometric methods and blood pressure measurements, and to evaluate its value for the detection of the MetS risk factor complex.

Therefore, the aim of this pilot study was to evaluate the reliability and validity of MetS (risk factor) diagnostics in a mobile diagnostic setup using various POC devices. First, blood analysis results obtained on-site in the mobile unit with three different POC analyzers were to be compared with those provided by a central clinical reference laboratory. Second, the test-retest-reliability and accuracy of diagnosis of MetS using POC analyzers and anthropometric measurements in the developed mobile examination unit should be evaluated.

## Methods

### Study design

This was a pilot reliability study in preparation for a regional cohort study on mobile diagnostics of MetS in the federal state of Brandenburg, Germany. The protocol of the entire study has been described elsewhere [23]. For the current pilot study, first, three POCs (Alere Cholestech LDX (Alere GmbH, Germany; “Alere”); Samsung LABGEO^PT10^ (Samsung Healthcare, South-Korea; “LABGEO”); CardioChek (PTS Diagnostics, USA; “CardioChek”) were compared against values measured in a reference laboratory for the most suitable POC in a mobile diagnostic setting. Secondly, the test-retest-reliability and accuracy of MetS diagnosis using the most suitable POC in a mobile diagnostic setting were assessed.

### Mobile diagnostic setting

**Fig. 1:**
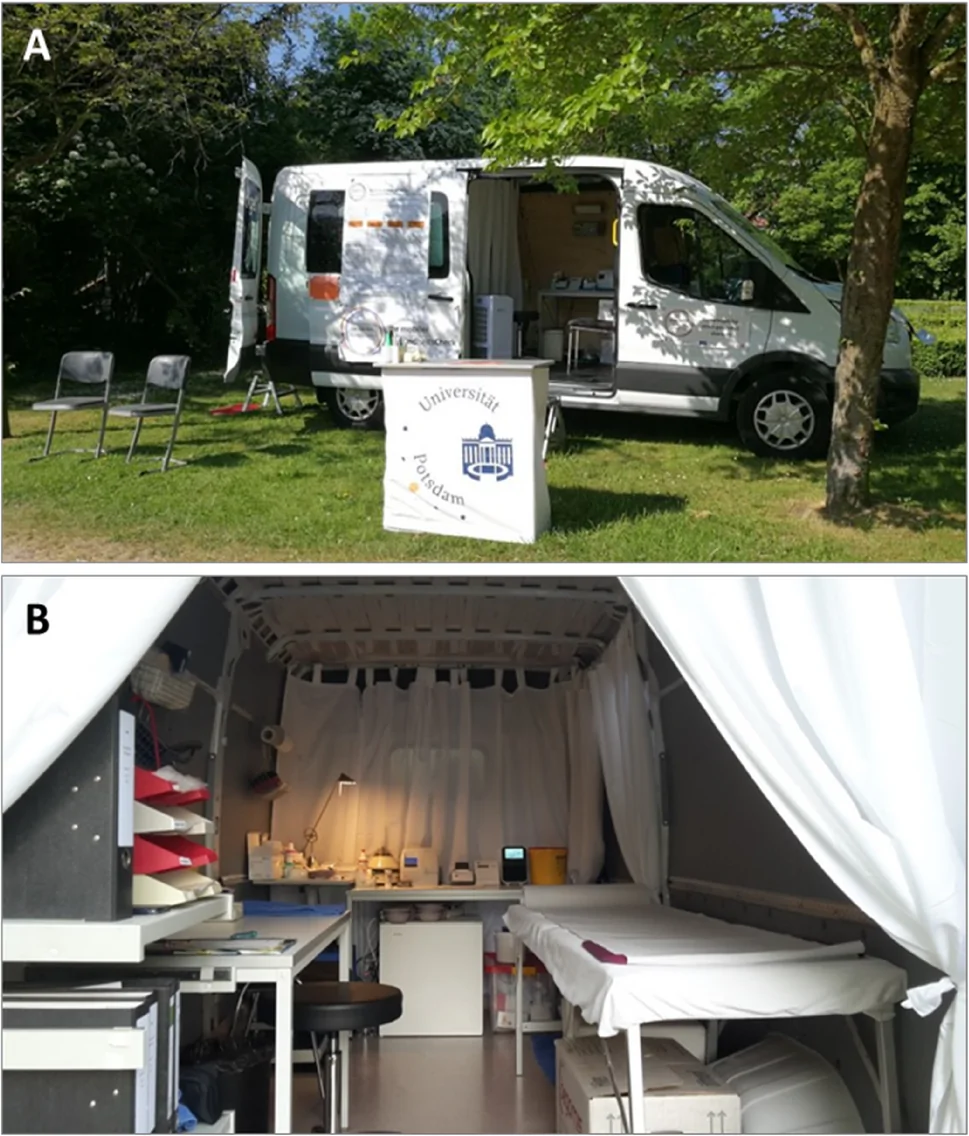
Mobile examination unit (**A**) equipped with tables, stools, shelves, curtains, laboratory materials and measuring instruments including portable point-of-care-analyzers (**B**)

In this pilot study, a mobile diagnostic unit was developed by setting up a van resembling a medical examination room. For this purpose, the van was equipped with tables, shelves, laboratory materials and measuring instruments, including portable POC analyzers, as well as seats for the participant and medical staff (Fig. 1). Curtains were installed to provide privacy. The entrance to the van was facilitated by a small staircase. Either portable heaters or portable air coolers were used to regulate room temperature. Power was available through external access to an electrical outlet.

This mobile diagnostic unit was strategically placed in front of shopping malls or health centres across rural areas in the state of Brandenburg (Germany). Participants were screened for MetS using three different POC blood analyzers. Results obtained by the portable devices were compared to results obtained by a central reference laboratory to select the analyzer with the most reliable results in the context of the mobile diagnostic unit. Participants received their test results immediately and were referred to a general practitioner for further diagnosis, if necessary, based on the MetS diagnosis and abnormal parameters. The entire MetS screening and assessment of all parameters were performed by a healthcare professional and took no longer than approximately 15 min. A second measurement was performed for the test-retest analysis within 2 weeks in between the measurements.

### Participants and recruitment

Participants (men and women) aged between 40 and 70 years were recruited from the general population in the federal state of Brandenburg, Germany. A passive recruitment approach was employed, with the target population being residents of Brandenburg who volunteered to participate in the mobile diagnostic unit on designated measurement days [24]. Recruitment was supported by local advertising with flyers and posters prior to the measurement days. The recruitment of participants took place in 2016.

Fifty participants (29 females, 21 males; age: 52 ± 7 years; height: 170 ± 10 cm; weight: 80 ± 19 kg) were enrolled for the pilot study. Participants were in a non-fasting state at the time of measurement due to the spontaneous recruitment. Individuals with MetS diagnosis using any of the devices at the first measurement day were asked to participate in a second measurement, no more than two weeks later. This test-retest analysis was conducted with a subgroup of 16 participants (male/female: 10/6). All participants gave their written informed consent. The study was approved by the ethics committee of the University of Potsdam (No. 40/2016).

### Procedures and outcomes

#### Medical history

After entering the van, participants were asked about pre-existing conditions and diseases, and current medication to treat type 2 diabetes mellitus, hypertriglyceridemia, hypercholesterolemia, and hypertension, which are inherent parts of the MetS diagnosis (Table 1).

#### Blood sampling and analysis

Capillary blood was obtained from the fingertip using safety lancets and stored in capillary tubes (70 µL). Blood glucose and blood lipids (HDL and triglycerides) were analyzed concurrently using three different commercially available and validated point-of-care (POC) analyzers: (1) Alere Cholestech LDX (Alere GmbH, Germany; “Alere”); (2) Samsung LABGEO^PT10^ (Samsung Healthcare, South-Korea; “LABGEO”); (3) CardioChek (PTS Diagnostics, USA; “CardioChek”). The measuring ranges for the blood analysis devices were for Alere: blood glucose: 50–500 mg/dl, triglycerides: 45–650 mg/dl, HDL: 15–100 mg/dl; for LABGEO: blood glucose: 70–110 mg/dl, triglycerides: 5–200 mg/dl, HDL: 40–75 mg/dl; and for CardioChek: blood glucose 40–600 mg/dl, triglycerides: 50–500 mg/dl, HDL: 15–100 mg/dl. For the measurement of HbA1c, the Quo-Test A1C Test Kit (EKF-diagnostic GmbH, Germany) [25] was used (measuring range: 4–15%). All POC devices provided the measurement results within approximately 5 min.

The values for blood glucose, triglycerides and HDL measured with the POC analyzers were eventually compared against values obtained in a clinical reference laboratory (“RefLab”). For this aim, additional blood samples were taken from an antecubital forearm vein using a disposable needle and a vacutainer. Blood samples were immediately centrifuged and stored cooled for the later analysis in an accredited external medical laboratory. The blood samples sent to the reference laboratory were generally analyzed within 12 h of collection.

#### Measurement of blood pressure and waist circumference

Systolic and diastolic blood pressure were measured using the auscultatory method with a manual sphygmomanometer and stethoscope. The participant had been in a seated position for 5 min and the blood pressure was measured mainly in the left arm. A non-elastic measuring tape (Seca 201, Germany) was used for measuring the waist circumference according to WHO guidelines (between the lowest rib and the top of the iliac crest of the bare upper body) [26].

#### Diagnosis of metabolic syndrome

MetS diagnosis was made according to the definition by Alberti et al. 2009 [1] (Table 1). Participants were not always in a fasted state shortly before the blood sampling due to spontaneous recruitment in the mobile setting during the day. Consequently, the cut-off point for elevated blood glucose was adjusted according to the paper of Schipf et al. [2]. It has been demonstrated that non-fasting lipid levels are also acceptable for estimating initial risk and diagnosing MetS [27]. Furthermore, the difference between fasting and non-fasting values is often small [28]. Therefore, the measurement of the lipid profile independent of the fasting status was included in the current study to test validity and reliability.

**Table 1:** Cut-off values and criteria for diagnosis of the metabolic syndrome according to Alberti et al., 2009 [1]

Metabolic syndrome is diagnosed in the presence of 3 of the 5 risk factors:	
Parameter	Cut-off values / criteria for classification as “risk factor”
Blood pressure	systolic ≥ 130 mmHg and/or diastolic ≥ 85 mmHg or antihypertensive drug treatment
Triglycerides	≥ 150 mg/dl (1,7 mmol/L) or drug treatment for elevated triglycerides
HDL-cholesterol	males: < 40 mg/dl (1,03 mmol/L), females: < 50 mg/dl (1,29 mmol/L) or drug treatment for reduced HDL-cholesterol
Blood glucose	≥ 100 mg/dl (fasting; 5,6 mmol/L) or ≥ 144 mg/dl (non-fasting)* or drug treatment for elevated glucose/type 2 diabetes mellitus
Waist circumference	males: ≥ 94 cm, females: ≥ 80 cm (population-/country-specific, according to IDF recommendation for the European population)

*Abbreviations*: *IDF:* International Diabetes Federation, *HDL:* High density lipoprotein

*Adaption to non-fasting blood samples according to Schipf et al., 2010 [2]

Results for each variable were classified as ‘risk’ or ‘no risk’ for the results obtained by each of the three POC devices and the reference laboratory. If the measurement results for a variable were at the upper or lower limit of the measurement range of the respective POC, they could not be quantified precisely. However, based on the result whether a value was at the upper or lower limit of a measurement range it was still possible to determine whether the value was above or below the thresholds to be classified as a MetS risk factor (Table 1).

In addition, the more stable and meal-independent glycated hemoglobin (HbA1c‘) was determined to get additional hints about impaired long-term regulation of glucose metabolism and to provide meaningful assessment of metabolic risk factors even in the non-fasting state [27, 29].

### Data analysis

Results from blood analysis with the POC analyzers and the reference laboratory were presented descriptively as mean ± standard deviation (SD) as well as median ± IQR. Measurement errors were recorded as “missing values”. Measurement results at the upper or lower limit of the measurement range of each device were not exactly quantifieable, but still evaluable for risk factor classification.

The agreement of blood glucose, triglycerides and HDL levels measured with three different point-of-care analyzers compared with results from a reference laboratory was analyzed. The number of evaluable measurements of each analyzer was recorded and the bias with limits of agreement (bias; 2.5th and 97.5th percentile) and a rank ICC were calculated. The agreement between the measuring devices and the reference laboratory were visualized by plotting the differences against the mean in Bland-Altman plots. Normality of data distribution was tested with the Shapiro-Wilk test. Due to a non-normal distribution of differences, the non-parametric approach for Bland-Altman plots was conducted [30]. Further, McNemar’s test was performed to detect significant differences in MetS diagnosis based on results of each POC analyzer compared with the reference laboratory values. The most accurate device according to the descriptive analyses and McNemar’s test was chosen for the analysis of the test-retest-reliability of the MetS diagnosis, using this particular device. Differences between the mean values of the parameters on measurement day 1 and day 2 were analyzed using the Wilcoxon signed rank test. The reliability analysis was performed by calculating Spearman’s *rho*, and a rank intraclass correlation coefficient (two way random, single measures) [31] and test-retest variability (TRV%). Validity of the most accurate device was assessed by Bland-Altman analysis (bias; LoA (2.5th and 97.5th percentile) and plots of the data at the test-retest timepoints.

Additionally, the agreement of HbA1c measured with a POC analyzer compared to outcomes from the reference laboratory measurements, and the test-retest reliability was determined. The level of significance was set at α = 0.05. The Bland-Altman plots were generated using Microsoft Excel, whereas inferential statistical analyses were conducted in IBM SPSS.

## Results

### POC analyzers vs. reference lab

In the 50 subjects, median (± IQR) blood pressure values were 125 ± 15 mmHg (systolic) and 80 ± 19 mmHg (diastolic) and median (± IQR) waist circumference was 99 ± 17 cm (men) and 83 ± 10 cm (women). For all analyzed blood parameters, LABGEO had the highest number of evaluable measurements (Table 2). The CardioChek device had the largest number of missing values due to measurement errors in the triglyceride level analyses (missing values out of 50 (triglycerides): RefLab: 1; Alere: 4; LABGEO: 1; CardioChek: 46). For the Alere device, two triglyceride and one HDL measurements were outside the measurement range of the instrument, and in LABGEO, five triglyceride and one HDL measurements could not be exactly quantified because they exceeded the measurement range of the device. Looking at the respective quantifiable readings, the measurements of blood glucose, triglycerides, and HDL with all three different POC analyzers yielded descriptively higher median values compared to the results of the reference laboratory measurements, except for triglycerides measured with Alere. Comparing the POCs, the LABGEO device provided the highest median values for blood glucose and triglycerides and the lowest for HDL. Due to the varying numbers of quantifiable measurements between the devices, no statistical tests comparing the three devices were performed. With respect to the reference laboratory, the measurements performed with the LABGEO analyzer showed the highest bias, the highest SEM and the lowest ICC for all three measured parameters (Table 2).

**Table 2:** Analysis of blood glucose, triglycerides and high-density lipoprotein cholesterol (HDL) in 50 blood samples by the Alere, LABGEO and CardioChek point-of-care (POC) analyzers compared to a reference laboratory (RefLab) and diagnosis of metabolic syndrome (MetS)

	Glucose (mg/dl)								Triglycerides (mg/dl)								HDL (mg/dl)								MetS diagnosis	
			Agreement POC – RefLab					# (§)			Agreement POC – RefLab					# (§)			Agreement POC – RefLab					# (§)	N	McNemar’s test [POC vs. RefLab]
	Mean ± SD	Median ± IQR	SEM	ICC	Bias	LoA			Mean ± SD	Median± IQR	SEM	ICC	Bias	LoA			Mean ± SD	Median± IQR	SEM	ICC	Bias	LoA				
						2.5	97.5							2.5	97.5							2.5	97.5			
Alere	113 ± 23	109 ± 20	3.8	0.858	-5	-25	6	46 (46)	175 ± 95	140 ± 140	3.2	0.948	8	-58	30	44 (46)	57 ± 18	58 ± 28	2.1	0.939	-2	-14	9	45 (46)	12/46	p > 0.05
LABGEO	128 ± 38	114 ± 35	20.7	0.574	-10	-106	4	49 (49)	255 ± 125	254 ± 176	85.9	0.510	-39	-369	15	44 (49)	46 ± 13	48 ± 21	5.6	0.827	9	-5	26	48 (49)	30/49	p < 0.05
CardioChek	116 ± 25	113 ± 24	4.9	0.837	-8	-31	10	46 (46)	175 ± 22	169 ± 18	60.0	-0.016	-2	-66	43	4 (4)	62 ± 18	56 ± 29	3.4	0.931	-5	-22	4	46 (47)	4/27	-^1^
Reference laboratory	106 ± 20	106 ± 19				-		48 (48)	172 ± 97	142 ± 97				-		49 (49)	56 ± 16	56 ± 24				-		49 (49)	14/48	-

Data is presented as mean ± standard deviation (SD); median ± IQR; Number of [#] quantifiable and [§] evaluable (risk factor classification) measurements from 50 samples; *SEM:* Standard error of measurement;*ICC:* Intraclass correlation coefficient (Rank); Bias represented as median of differences; *LoA*: Limits of Agreement (2.5^th^ and 97.5^th^ percentile); *N:* Number of participants diagnosed with MetS out of number of data sets evaluable for MetS. McNemar’s test: difference in MetS diagnosis using each Point-of-care analyzer vs. reference lab, α = 0.05

^1^Too many missing data points to perform McNemar’s test for CardioChek

**Fig. 2:**
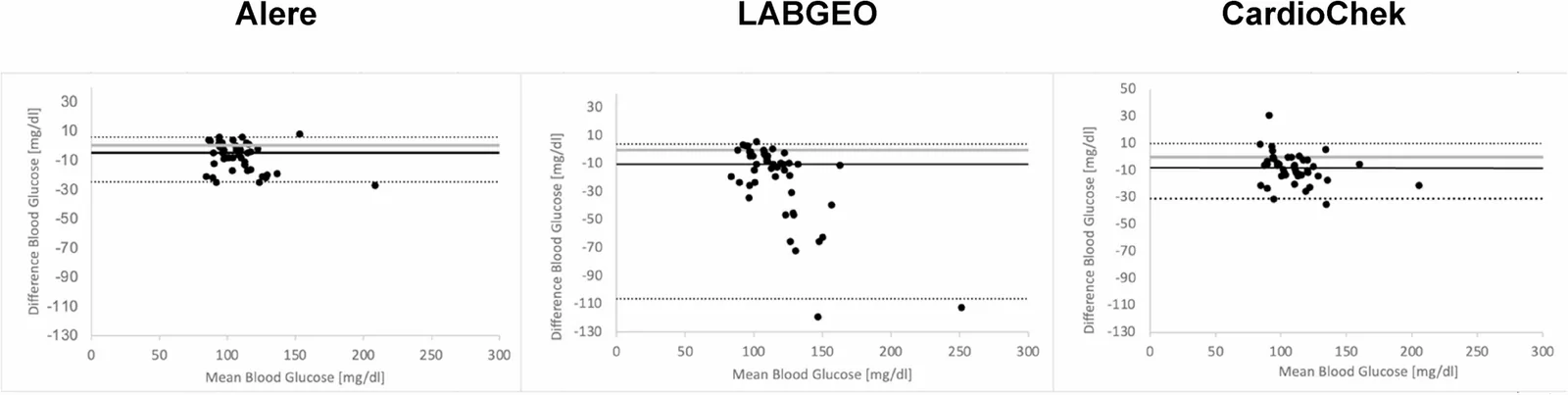
Bland-Altman plots of blood glucose measurement by three different point-of care analyzers (Alere, LABGEO and CardioChek) compared to reference lab measurements. In each graph, the solid black line represents the bias, the two dotted lines represent the lower and upper limits of agreement; the solid grey line represents reference lines (bias = 0)

**Fig. 3:**
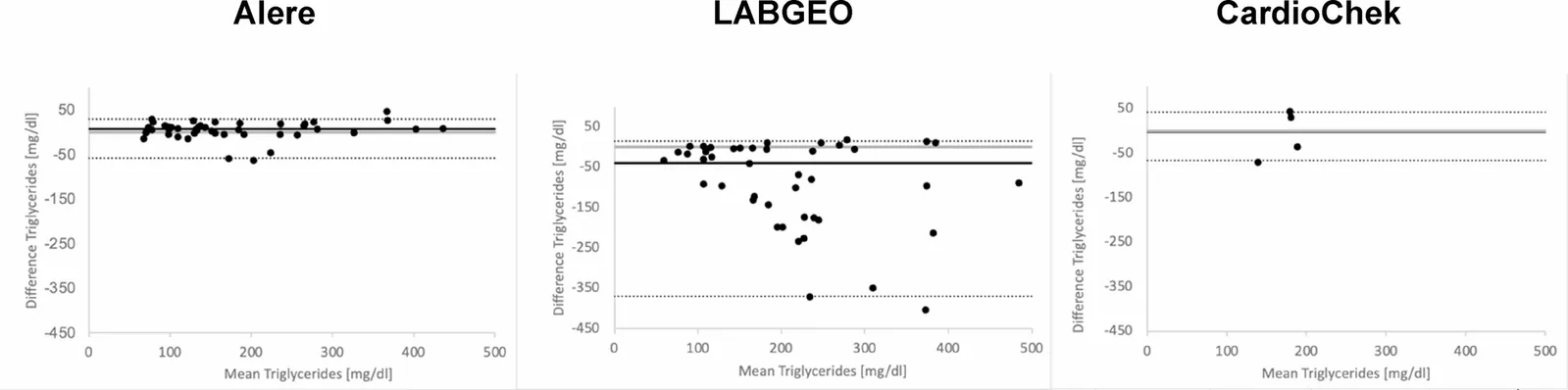
Bland-Altman plots of triglycerides measurement by three different point-of care analyzers (Alere, LABGEO and CardioChek) compared to reference lab measurements. In each graph, the solid black line represents the bias, the two dotted lines represent the lower and upper limits of agreement; the solid grey line represents reference lines (bias = 0)

**Fig. 4:**
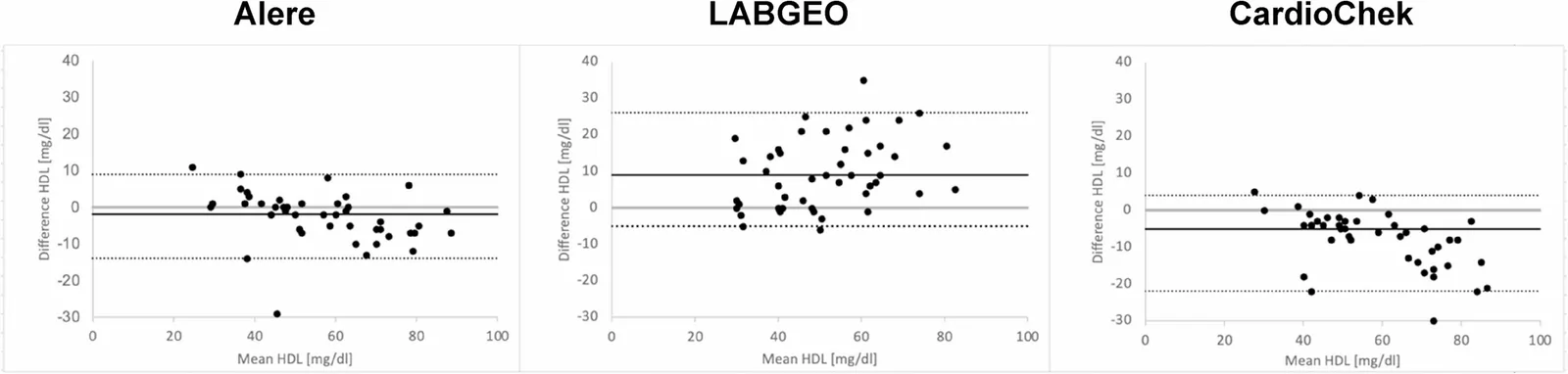
Bland-Altman plots of HDL measurement by three different point-of care analyzers (Alere, LABGEO and CardioChek) compared to reference lab measurements. In each graph, the solid black line represents the bias, the two dotted lines represent the lower and upper limits of agreement; the solid grey line represents reference lines (bias = 0)

As can be seen from the Bland-Altman plots (Figs. 2, 3 and 4), the majority of data points for all three POC devices were within the limits of agreement. The device from Alere showed the smallest range of bias (blood glucose: -5 [LoA: -25, 6] mg/dl; triglycerides: 8 [LoA: -58, 30] mg/dl; HDL: -2 [LoA: -14, 9] mg/dl). The Bland-Altman plot visually showed a slight proportional bias with higher differences in the lower measurement range for the HDL measurement in both Alere and CardioChek (Fig. 4). Due to the large number of missing values, CardioChek was excluded from further analyses regarding MetS diagnosis. The Alere device showed the best agreement and no significant statistical difference in the number of participants diagnosed with MetS based on data obtained with the POC analyzer compared to the results of the reference laboratory (MetS diagnosis (n out of evaluable measurements): RefLab, *n* = 14 (out of 48); Alere, *n* = 12 (out of 46); LABGEO, *n* = 30 (out of 49); McNemar’s test: Alere vs. RefLab: *p*>0.05; LABGEO vs. RefLab: *p* < 0.05).

**Fig. 5:**
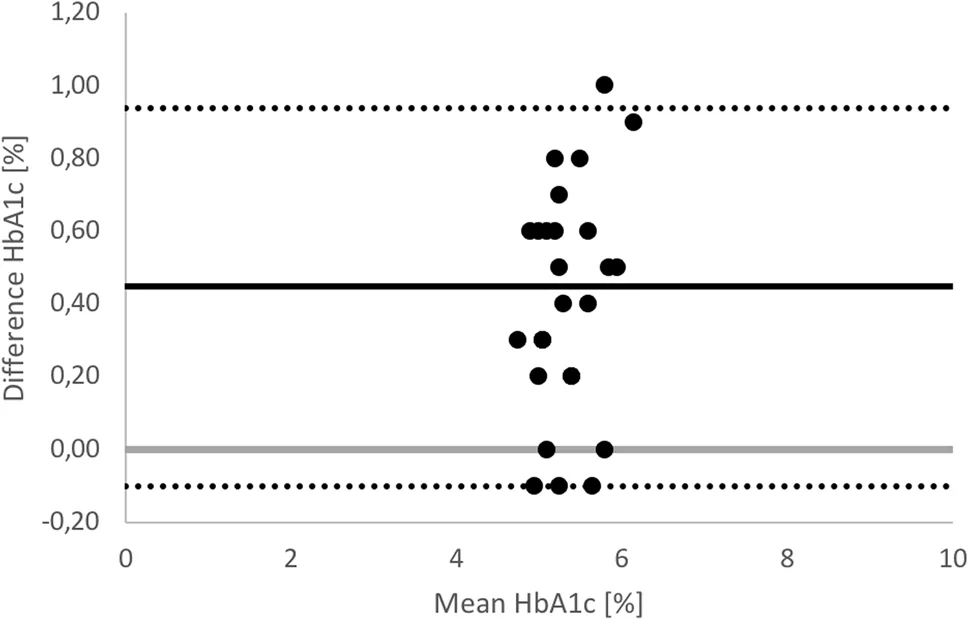
Bland-Altman plot comparing the measurement of HbA1c (%) using the Quo-Test A1C Test Kit to reference lab (RefLab) measurements

Only 26 out of 50 HbA1c‘ measurements with the Quo-Test A1C Test Kit were evaluable. The median value of these measurements with the POC analyzer (5.5 ± 0.6%, *n* = 26) was descriptively higher than the median of the reference laboratory analyses of the same participants (5.1 ± 0.4%, *n* = 26). The comparison of the evaluable HbA1c measurements with the Quo-Test A1C Test Kit and by a reference laboratory resulted in a bias of 0.45% with LoA of [-0.10, 0.94] %. There was no visually detectable proportional bias in the Bland-Altman plot (Fig. 5).

The categorization of HbA1c‘ as an indication of hyperglycemia (≥ 5.7% [32]) was not significantly statistically different between the measurement results obtained with the POC analyzer compared to those of the reference laboratory (Hyperglycemia: QuoTest, *n* = 8; RefLab, *n* = 4; McNemar’s test, *p* > 0.05).

### Test-retest analysis

**Fig. 6:**
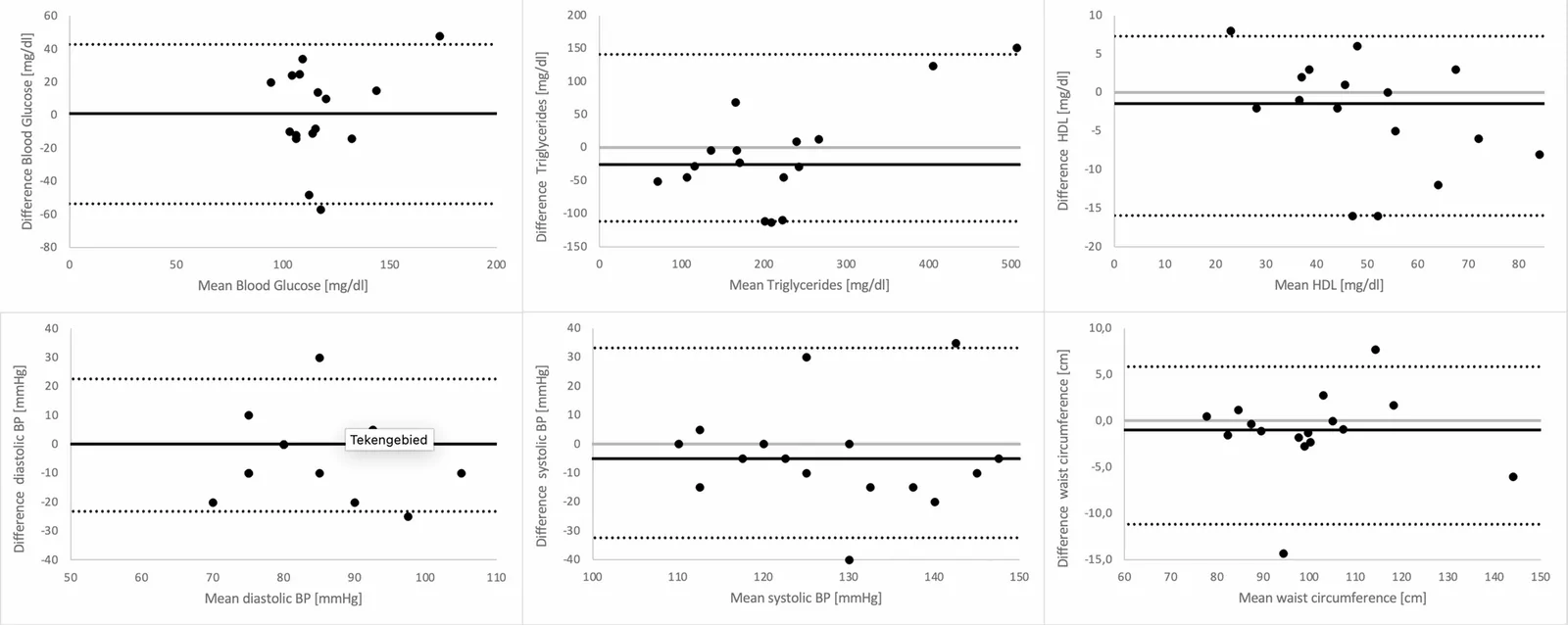
Bland-Altman plots assessing the agreement between day 1 and day 2 of the MetS risk factors (blood glucose (**A**), triglycerides (**B**), HDL (**C**), diastolic BP (**D**), systolic BP (**E**), and waist circumference (**F**)) measured by the Alere Cholestech LDX point-of-care analyzer

**Fig. 7:**
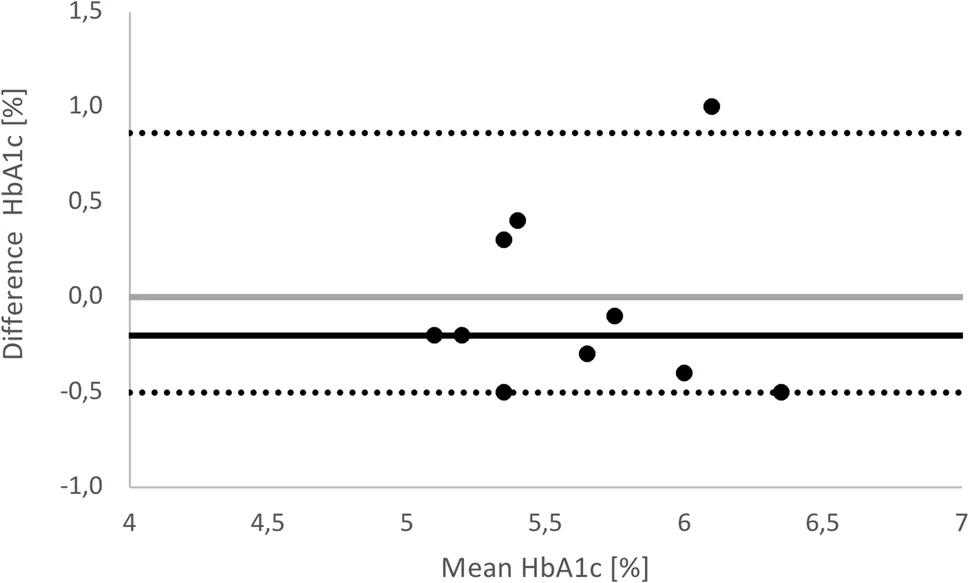
Bland-Altman plots assessing the agreement between day 1 and day 2 of HbA1c, measured by the Quo-Test A1C Test Kit

The test-retest analysis of blood parameter measurement and MetS diagnosis was performed using the POC devices Alere and Quo-test in the mobile setting with a subset of 16 participants. The mean values for each parameter didn’t differ statistically significant (Wilcoxon signed rank test, *p* > 0.05) between both measurement days (Table 3). Overall, the Spearman’s *rho* ranged from *r =* 0.078 to *r* = 0.938 the ICC from 0.083 to 0.970, and TRV from 0.9% to 5.8%. The lowest ICC and spearmans *rho* belonged to the blood glucose analyses and the blood pressure measurement. Bland-Altman plots assessing the agreement of the measurements at day 1 and day 2 are shown in Fig. 6 (Alere) and Fig. 7 (Quo-test). The values for all parameters were within the limits of agreement. No inter-session differences for each risk factor classification and the overall MetS diagnosis were shown (McNemar’s test day 1 vs. day 2: *p *> 0.05).

**Table 3:** Test-retest analysis of MetS diagnosis using the Alere point-of-care analyzer and of Hb1Ac‘ measurements using the Quo-Test device in a mobile setting with 16 participants.

	Day 1		Day 2		Wilcoxon signed rank test		Spearman’s rho	ICC	TRV	Bias	LoA		MetS risk factor classification		
	Mean ± SD	Median ± IQR	Mean ± SD	Median ± IQR	Z	*p*					2.5^th^	97.5^th^	day 1 [n]	day 2 [n]	McNemar‘s test
Blood glucose	117 ± 20	114 ± 31	118 ± 27	114 ± 25	79	0.569	0.078	0.083	0.9%	1	-54	43	2	2	*p* > 0.05
(mg/dl)															
Triglycerides	221 ± 90	240 ± 125	209 ± 138	166 ± 89	49	0.326	0.734*	0.747*	5.8%	-26	-112	141	11	11	*p* > 0.05
(mg/dl)															
HDL	51 ± 18	50 ± 25	48 ± 15	45 ± 16	40	0.243	0.893*	0.899*	5.6%	-1.5	-16	7	5	6	*p* > 0.05
(mg/dl)															
Diastolic blood pressure	88 ± 12	85 ± 13	84 ± 12	80 ± 11	15	0.196	0.442	0.458	4.4%	0	-23	23	11	10	*p* > 0.05
(mmHg)															
Systolic blood pressure	130 ± 15	128 ± 26	126 ± 15	123 ± 18	26	0.16	0.394	0.41	3.4%	-5	-33	33			
(mmHg)															
Waist circumference	101 ± 16	101 ± 16	100 ± 16	98 ± 18	41	0.281	0.938*	0.970*	1.1%	-1	-11	6	13	13	*p* > 0.05
(cm)															
MetS diagnosis	-	-	-	-			-	-	-	-			8	8	*p* > 0.05
HbA1c (%)^#^	5.7 ± 0.5	5.6 ± 0.6	5.6 ± 0.5	5.6 ± 0.6	21	0.507	0.59	0.615	0.9%	-0.2	-0.5	0.9	-	-	-

Data is presented as mean ± standard deviation (SD); Median ± IQR; *ICC*: Intraclass correlation coefficient (Rank); *TRV:* Test-retest variability; Bias: median of differences; *LoA:* Limits of Agreement (2.5th and 97.5th percentile); [n]: number of participants with prevalent risk factor at each day (from N=16). McNemar’s test: difference in risk factor classification and MetS diagnosis, day 1 vs. day 2, α = 0.05. #: N=10, due to missing values at day 1; prevalence and McNemar’s test outcomes for HbA1c are not presented, since this parameter was not part of MetS diagnosis. (*)Statistically significant (*p *< 0.05) result

## Discussion

In this pilot study, a setup was developed to enable mobile and rapid screening for MetS. Among the point-of-care analyzers evaluated in the (mobile) setting of this study, the Alere Cholestech LDX was the most accurate portable device with the smallest bias, a low number of missing values and the highest agreement of results compared with those of the reference laboratory. The results of the blood analysis with the Alere device resulted in a comparable MetS diagnosis to the reference laboratory results, even though this device also showed its limitations regarding test-retest reliability for the blood glucose parameter. Overall, the mobile screening setup with the selected POC blood analyzer was therefore valid to diagnose MetS within 15 min in an on-site mobile examination unit to ensure early detection of risk factors.

In a study directly comparing the devices CardioChek and Alere, a smaller percentage of bias and higher ICCs (all > 0.75) for the parameters total cholestrol, HDL-C, low-density lipoprotein cholestrol and triglycerides were shown for Alere Cholestech LDX [33]. In comparison, the CardioChek device had lower reproducibility, and the measured parameters led to a higher misclassification of risk compared to the results obtained with a reference laboratory. Although the CardioChek device also had high ICCs for blood glucose and HDL measurements in the current study, the large number of invalid triglyceride measurements made the device unusable in our mobile diagnostic setting. The large number of missing values for the triglyceride measurements were partially due to an error in the device and to an insufficient amount of blood to conduct the analysis with the CardioChek device. Nonetheless, even if more evaluable measurements would have been available for the CardioChek device, it is unlikely that this would have altered the decision to continue the test-retest with the Alere device, as the bias for the two other components measured with a POC compared to a reference laboratory was lower for the Alere versus the CardioChek device (blood glucose Alere: -5 mg/dl vs. CardioChek: − 8 mg/dl; HDL Alere: -2 mg/dl vs. CardioChek: -5 mg/dl).

For the LABGEO device, analyses of capillary blood samples have previously been shown to give comparable results to those obtained in a central reference laboratory [34]. Lim et al. 2017 showed lower bias (blood glucose: 5.7 mg/dl, triglycerides 0.1 mg/dl, HDL: 6.8 mg/dl) than we calculated in our analyses. In the current mobile diagnostic setting, the LABGEO device showed higher bias than in the study of Lim et al. 2017, especially for triglycerides (blood glucose: -10 mg/dl, triglycerides: − 39 mg/dl, HDL: 9 mg/dl). To our knowledge, there is no study directly comparing the Alere and LABGEO devices. There are several possible reasons why the LABGEO device did not perform as well in the current setting, such as sensitivity to environmental influences (e.g. termperature), calibration issues and others. The precise cause of the device’s inaccurate performance in the current study remains undetermined. Alere showed the most accurate results and no statistically significant differences in MetS diagnosis compared to the reference laboratory. The small number of missing values was due to sensitivity to ambient temperature. To minimize these measurement errors, care must be taken in the mobile setting to keep the temperature of the environment and material constant. Depending on the season, air coolers or heaters should be used for this purpose.

The test-retest variability for the Alere device was low for all evaluated parameters (0.9% − 5.8%). The test-retest reliability was poor for blood glucose, poor to moderate for blood pressure, moderate to good for triglycerides and good to excellent for both HDL and waist circumference (Table 3) [31]. However, the poor or moderate test-retest reliability for blood glucose and blood pressure did not impact the overall MetS diagnosis, as shown by the McNemar’s test (day 1 vs. day 2: *p* > 0.05). Even though the Alere POC is the most suitable out of the three POC’s tested, it still has a bias compared to the reference lab and for full certainty, a full screening in the reference lab is prefered. However, as a POC allows for testing ‘in the field’ and people in rural areas commonly have a more limited access to healthcare, it is recommended for initial testing.

The poor to moderate test-retest reliability of the blood pressure measurements could be explained by several factors known to cause high variability between multiple measurements, such as physical activity, time of measurement, circadian rhythm, and drug intake [35]. Furthermore, variations in blood pressure can be caused by the so called “white coat syndrome”, meaning a difference in blood pressure can occur depending on the person performing the examination (health care professional (clinical setting) vs. self (home)), with a blood pressure elevation present when measured by medical staff [36]. Due to the unfamiliar situation, especially on the first day of measurement, the nervousness of some participants might have influenced the blood pressure measurement. Ten out of the 16 participants had lower systolic blood pressure values on the second measurement day, but despite these variations, values were still above or below the cutoff points, and considering existing medication, only one person’s risk factor classification changed. In this case, overall MetS diagnosis was not influenced.

Blood glucose also demonstrated poor reliability, which may have been due to the varying times at which this parameter was measured as a result of spontaneous recruitment, as well as whether participants were in a fasting state. The low test-retest ICC and *r* of blood glucose did not significantly affect the diagnosis of MetS among the selected sample. One possibility for the low influence of blood glucose on the MetS diagnosis could be that this risk factor is less commonly present in our sample of participants than the other factors. Blood glucose as a risk factor for MetS was present in 2/16 participants on day one and 2/16 participants on day two. The other risk factors (day 1 vs. day 2: HDL, 5/16 vs. 6/16; triglycerides, 11/16 vs. 11/16; blood pressure, 11/16 vs. 10/16; waist circumference, 13/16 vs. 13/16) showed a much higher prevalence in the assessed population. The idea that blood glucose doesn’t influence MetS diagnosis as much as other factors is supported in the study of Schipf et al. (2010), who screened a population of 4223 participants with German citizenship (aged 20–79 years) for MetS and found that blood glucose was the risk factor with the lowest prevalence (6.2%) [2]. Since there is such a low predictable value of blood glucose for MetS, the possibility to replace this factor with the more stable HbA1c as a better marker for MetS should be assessed as proposed previously [27, 29]. In the current study, the Hb1Ac‘ measurement with Quo-Test POC analyzer compared to the reference laboratory resulted in a low bias of 0.45%, with a small range of LoA. In contrast, previous studies comparing this POC analyzer with laboratory methods often showed negative bias [25]. The test-retest measurements for HbA1c resulted in a moderate reliability and a low variability (Table 3). Unfortunately, there was a high amount of missing data for this variable, due to very high temperature sensitivity of the Quo Test point-of-care analyzer in our measurement set-up. Therefore, this particular analysis device has limited suitability in our mobile setting and could only serve as an optional measurement to provide additional information on an impaired glucose metabolism.

The study of Després (2006) noted that the most prevalent form of MetS that physicians will come across is related to abdominal obesity, indicating that waist circumference is a prominent risk factor among MetS patients [37]. This notion is confirmed in our results (prevalence waist circumference day 1 & 2: 13/16) and the study of Schipf et al. (2010), who noted increased waist circumference as the most prevalent (49.4%) risk factor after blood pressure, indicating a high influence on MetS diagnosis.

### Clinical implications

The results of this study show that MetS diagnosis with a mobile examination unit using the Alere Cholestech LDX device as a point-of-care analyzer could be a suitable alternative for initial diagnosis using reference laboratory measurements in rural areas with limited access to health care providers. The total examination in the mobile unit requires approximately 15 min and is therefore not time consuming. Implementing vans as examination units, as was done in this study, could increase the number of people that can be screened for MetS, to detect risk factor profiles before the onset or manifestation of secondary diseases. Individuals at risk could, if necessary, be referred to a qualified healthcare professional and adequate prevention programs could be implemented. This strategy could help to prevent the development of secondary diseases and therefore lowering the disease burden as well as economic burden.

### Strengths & limitations

According to our knowledge, this is the first study comparing various point-of-care analyzers for MetS analysis in a mobile setting, therefore aiming to simplify MetS check-ups which could be used for fast risk factor screening.

Because of the study design, which involves spontaneous recruitment, it was not possible to ensure that participants were in a fasting state. Although it has been suggested that nonfasting lipids could also be used for risk factor classification and MetS diagnosis, this is a limitation, at least until the MetS definition is revised or expanded with respect to nonfasting parameters or supplemented with more stable variables. Nevertheless, the mobile diagnostic approach is appropriate for initial screening to identify at-risk individuals. Abnormal results should then be reviewed by a physician during a thorough examination.

Another limitation of this study is the non-standardized time between MetS risk factor measurements for the test-retest calculations. Even though there was a maximum of two weeks between the measurements, there is no mandatory number of days that all participants had to have between measurements.

Lastly, the sample size for the test-retest calculation was limited and focused on people with MetS. Ideally, the results of the main study should confirm or deny the test-retest reliability results in a bigger sample, possibly also broadly in the general population. Nonetheless, the test-retest analysis of this pilot study provides important implications for future studies and adds to the evidence base regarding POCs, as, according to the authors’ knowledge, this is the first study conducting test-retest analyses on two different days with regards to metabolic syndrome.

## Conclusions

The results of this study indicate that the mobile examination unit equipped with the Alere Cholestech LDX blood analysis device is an accurate and fast method for a near-to-home screening for MetS to identify persons at risk, for example among rural areas with poor access to health care providers. However, the low reliability for blood glucose measurement could possibly affect MetS diagnosis, although it did not affect this study’s outcomes.

## Data Availability

The datasets used and/or analyzed during the current study are available from the corresponding author upon reasonable request.

## Abbreviations

BP: Blood pressure
Glc: Blood glucose
Hb1Ac‘: Glycated hemoglobin
HDL: High-density lipoprotein
ICC: Intraclass correlation coefficient
LoA: Limits of Agreement
MetS: Metabolic syndrome
POC: Point-of-care blood analyzers
RefLab: Clinical reference laboratory
SD: Standard deviation
SEM: Standard error of the mean
TG: Serum triglycerides
TRV: Test-retest variability

## References

[1] Alberti KGMM, Eckel RH, Grundy SM, Zimmet PZ, Cleeman JI, Donato KA, et al. Harmonizing the metabolic syndrome: A joint interim statement of the international diabetes federation task force on epidemiology and prevention; National heart, lung, and blood institute; American heart association; World heart federation; International. Circulation. 2009;120(16):1640–5. 10.1161/CIRCULATIONAHA.109.192644. 19805654 10.1161/CIRCULATIONAHA.109.192644

[2] Schipf S, Alte D, Völzke H, Friedrich N, Haring R, Lohmann T, et al. Prävalenz des Metabolischen Syndroms in Deutschland: Ergebnisse der Study of Health in Pomerania (SHIP). Diabetol und Stoffwechsel. 2010;5(3):161–8. 10.1055/s-0030-1247406.

[3] Srikanthan K, Feyh A, Visweshwar H, Shapiro JI, Sodhi K. Systematic review of metabolic syndrome biomarkers: A panel for early detection, management, and risk stratification in the West Virginian population. Int J Med Sci. 2016;13:25–38. 10.7150/ijms.13800. 26816492 10.7150/ijms.13800 PMC4716817

[4] Yamaoka K, Tango T. Effects of lifestyle modification on metabolic syndrome: A systematic review and meta-analysis. BMC Med. 2012;10:1–10. 10.1186/1741-7015-10-138. 23151238 10.1186/1741-7015-10-138 PMC3523078

[5] O’Neill S, O’Driscoll L. Metabolic syndrome: A closer look at the growing epidemic and its associated pathologies. Obes Rev. 2015;16(1):1–12. 10.1111/obr.12229. 25407540 10.1111/obr.12229

[6] Wang HH, Lee DK, Liu M, Portincasa P, Wang DQH. Novel insights into the pathogenesis and management of the metabolic syndrome. Pediatr Gastroenterol Hepatol Nutr. 2020;23(3):189–230. 10.5223/PGHN.2020.23.3.189. 32483543 10.5223/pghn.2020.23.3.189 PMC7231748

[7] Myers J, Kokkinos P, Nyelin E. Physical activity, cardiorespiratory fitness, and the metabolic syndrome. Nutrients. 2019;11:1–18. 10.3390/nu11071652. 10.3390/nu11071652 PMC6683051 31331009

[8] Eckel RH, Grundy SM, Zimmet PZ. The metabolic syndrome. Lancet. 2005;365(9468):1415–28. 10.1016/S0140-6736(05)66378-7. 15836891 10.1016/S0140-6736(05)66378-7

[9] Saklayen MG. The Global Epidemic of the Metabolic Syndrome. Curr Hypertens Rep. 2018;20:1–8. 10.1007/s11906-018-0812-z. 29480368 10.1007/s11906-018-0812-z PMC5866840

[10] Moebus S, Hanisch J, Bramlage P, Lösch C, Hauner H, Wasem J, et al. Regional differences in the prevalence of the metabolic syndrome in primary care practices in Germany. Dtsch Arztebl. 2008;105(12):207–13. 10.3238/artzebl.2008.0207. 10.3238/artzebl.2008.0207 PMC2696781 19629210

[11] Noubiap JJ, Nansseu JR, Nyaga UF, Ndoadoumgue AL, Ngouo AT, Tounouga DN, et al. Worldwide trends in metabolic syndrome from 2000 to 2023: a systematic review and modelling analysis. Nat Commun. 2026;17(1):1–13 . 10.1038/s41467-025-67268-5. 10.1038/s41467-025-67268-5 PMC12808104 41350289

[12] Chong KS, Chang YH, Yang CT, Chou CK, Ou H, Kuo S. Longitudinal economic burden of incident complications among metabolic syndrome populations. Cardiovasc Diabetol. 2024;23:1–10. 10.1186/s12933-024-02335-7. 38987782 10.1186/s12933-024-02335-7 PMC11238381

[13] Stuckey M, Fulkerson R, Read E, Russell-Minda E, Munoz C, Kleinstiver P, et al. Remote monitoring technologies for the prevention of metabolic syndrome: The Diabetes and Technology for Increased Activity (DaTA) study. J Diabetes Sci Technol. 2011;5(4):936–44. 10.1177/193229681100500417. 21880237 10.1177/193229681100500417 PMC3192601

[14] Goffrier MA, B LLM, Bätzing MPH, Dr med J, Holstiege MPHJ. Entwicklung der administrativen Prävalenz des Diabetes mellitus von 2009 bis 2015. Monit Versorgungsforschung. 2017;10(05):46–9. 10.24945/mvf.05.17.1866-0533.2040.

[15] Heidemann C, Kuhnert RBS. RKI - 12-Monats-Prävalenz des bekannten Diabetes mellitus in Deutschland - Fact sheet. J Health Monit. 2017;2(1):48–56. 10.17886/RKI-GBE-2017-008. 37151307 10.17886/RKI-GBE-2017-017 PMC10161271

[16] Stentzel U, Piegsa J, Fredrich D, Hoffmann W, Van Den Berg N. Accessibility of general practitioners and selected specialist physicians by car and by public transport in a rural region of Germany. BMC Health Serv Res. 2016;16(1):1–10. 10.1186/s12913-016-1839-y. 27756338 10.1186/s12913-016-1839-y PMC5070365

[17] Kuhn B, Kleij KS, Liersch S, Steinhäuser J, Amelung V. Which strategies might improve local primary healthcare in Germany? An explorative study from a local government point of view. BMC Fam Pract. 2017;18:1–12. 10.1186/s12875-017-0696-z. 29262798 10.1186/s12875-017-0696-z PMC5738820

[18] Schröder L, Flägel K, Goetz K, Steinhäuser J. Mobility concepts and access to health care in a rural district in Germany: A mixed methods approach. BMC Fam Pract. 2018;19:1–10. 10.1186/s12875-018-0733-6. 29720091 10.1186/s12875-018-0733-6 PMC5932842

[19] Yoon NH, Lee HY, Kwak MS, Choi KS, Jun JK, Kim MK, et al. Comparison of satisfaction with cancer screening at mobile van and static sites: National cancer screening program in Korea. Jpn J Clin Oncol. 2009;39(3):169–74. 10.1093/jjco/hyn156. 19179364 10.1093/jjco/hyn156

[20] Stanley E, Lewis MC, Irshad A, Ackerman S, Collins H, Pavic D, et al. Effectiveness of a mobile mammography program. Am J Roentgenol. 2017;209(6):1426–9. 10.2214/AJR.16.17670. 28871806 10.2214/AJR.16.17670

[21] Gopalan HS, Haque I, Ahmad S, Gaur A, Misra A. Diabetes care at doorsteps: A customised mobile van for the prevention, screening, detection and management of diabetes in the urban underprivileged populations of Delhi. Diabetes Metabolic Syndrome: Clin Res Reviews. 2019;13(6):3105-12. 10.1016/j.dsx.2019.11.008. 10.1016/j.dsx.2019.11.008 31790964

[22] Luppa PB, Müller C, Schlichtiger A, Schlebusch H. Point-of-care testing (POCT): Current techniques and future perspectives. TRAC Trends Anal Chem. 2011;30(6):887–98. 10.1016/j.trac.2011.01.019. 10.1016/j.trac.2011.01.019 PMC7125710 32287536

[23] Schraplau A, Block A, Häusler A, Wippert PM, Rapp MA, Völler H, et al. Mobile diagnostics and consultation for the prevention of the metabolic syndrome and its secondary diseases in Brandenburg—study protocol of a regional prospective cohort study: the Mobile Brandenburg Cohort. Pilot Feasibility Stud. 2021;7(1):1-11. 10.1186/s40814-021-00898-w. 34462012 10.1186/s40814-021-00898-w PMC8403821

[24] Lee RE, McGinnis KA, Sallis JF, Castro CM, Chen AH, Hickmann SA. Active vs. passive methods of recruiting ethnic minority women to a health promotion program. Ann Behav Med. 1997;19(4):378–84. 10.1007/BF02895157. 9706365 10.1007/BF02895157

[25] Hirst JA, McLellan JH, Price CPEE, Feakins BG, Stevens RJ, et al. Performance of point-of-care HbA1c test devices: implications for use in clinical practice – a systematic review and meta-analysis. Clin Chem Lab Med (CCLM). 2017;55(2):167–80. 10.1515/cclm-2016-0303. 27658148 10.1515/cclm-2016-0303

[26] World Health Organization. Waist Circumference and Waist-Hip Ratio. Report of a WHO Expert Consultation. 2008 Dec. 10.1038/ejcn.2009.139.

[27] Driver SL, Martin SS, Gluckman TJ, Clary JM, Blumenthal RS, Stone NJ. Fasting or Nonfasting Lipid Measurements. J Am Coll Cardiol. 2016;67(10):1227–34. 10.1016/j.jacc.2015.12.047. 26965545 10.1016/j.jacc.2015.12.047

[28] Langsted A, Nordestgaard BG. Nonfasting versus fasting lipid profile for cardiovascular risk prediction. Pathology. 2019;51(2):131–41. 10.1016/j.pathol.2018.09.062. 30522787 10.1016/j.pathol.2018.09.062

[29] Park SH, Yoon JS, Won KC, Lee HW. Usefulness of glycated hemoglobin as diagnostic criteria for metabolic syndrome. J Korean Med Sci. 2012;27(9):1057–61. 10.3346/jkms.2012.27.9.1057. 22969252 10.3346/jkms.2012.27.9.1057 PMC3429823

[30] Bland JM, AltmanDG. Measuring agreement in method comparison studies. Stat Methods Med Res. 1999;8(2):135–60. 10.1177/096228029900800204 . PMID:10501650 10.1177/096228029900800204 10501650

[31] Koo TK, Li MY. A Guideline of Selecting and Reporting Intraclass Correlation Coefficients for Reliability Research. J Chiropr Med. 2016;15(2):155–63. 10.1016/j.jcm.2016.02.012. 27330520 10.1016/j.jcm.2016.02.012 PMC4913118

[32] Schleicher E, Gerdes C, Petersmann A, Müller-Wieland D, Müller UA, Freckmann G, et al. Definition, Klassifikation und Diagnostik des Diabetes mellitus: Update 2021. Der Diabetologe. 2022;18:41–8. 10.1055/A-1515-8638.

[33] Dale RA, Jensen LH, Krantz MJ. Comparison of Two Point-of-Care Lipid Analyzers for Use in Global Cardiovascular Risk Assessments. Ann Pharmacother. 2008;42(5):633–9. 10.1345/aph.1K688. 18413684 10.1345/aph.1K688

[34] Lim J, Kim H, Koo SH, Kwon GC. Evaluation of the LABGEO PT10 Point-Of‐Care Testing Device: Comparison of Analyte Measurements in Capillary Whole Blood and Lithium Heparin Whole Blood Samples With Those in Central Laboratory. J Clin Lab Anal. 2017;31(3):1-11. 10.1002/jcla.22050. 10.1002/jcla.22050 PMC6817124 27638131

[35] Spallone V. Blood Pressure Variability and Autonomic Dysfunction. Curr Diab Rep. 2018;18:1–14. 10.1007/s11892-018-1108-z. 30361834 10.1007/s11892-018-1108-z

[36] Pioli MR, Ritter AMV, de Faria AP, Modolo R. White coat syndrome and its variations: differences and clinical impact. Integr Blood Press Control. 2018;11:73–9. 10.2147/IBPC.S152761. 30519088 10.2147/IBPC.S152761 PMC6233698

[37] Després JP. Abdominal obesity: The most prevalent cause of the metabolic syndrome and related cardiometabolic risk. Eur Heart J Supplement. 2006;8:4–12. 10.1093/eurheartj/sul002.

